# Short‐Term Effects of Folate Supplementation in Combination With Vitamin B6 for Treating Acute Manic Episodes in Bipolar I Disorder: A Randomized Controlled Trial

**DOI:** 10.1002/brb3.70432

**Published:** 2025-04-09

**Authors:** Farzad Akbarzadeh, Andisheh Talaei, Mohsen Nematy, Dina Ganji, Alireza Ebrahimi, Ali Talaei

**Affiliations:** ^1^ Psychiatry and Behavioral Sciences Research Center Mashhad University of Medical Sciences Mashhad Iran; ^2^ Faculty of Biotechnology Tehran University Tehran Iran; ^3^ Department of Nutrition, Faculty of Medicine Mashhad University of Medical Sciences Mashhad Iran

**Keywords:** bipolar I disorder, folate, mania, sodium valproate, vitamin B6

## Abstract

**Background:**

Drug resistance poses a formidable challenge in managing acute manic episodes in bipolar I disorder, leading to suboptimal treatment outcomes. This study investigates the efficacy of folate and vitamin B6 supplementation as an adjunct to sodium valproate in improving treatment responses for patients experiencing acute mania.

**Methods:**

In a randomized, double‐blind, placebo‐controlled trial, 43 patients diagnosed with bipolar I disorder presenting with acute manic episodes were enrolled. Participants were randomly assigned to three groups: one receiving folate (5 mg/day) plus vitamin B6 (80 mg/day), a second group receiving folate alone (5 mg/day), and a third group receiving placebo. Evaluations were conducted at baseline and after 3 and 6 weeks using the Mini‐Mental State Examination (MMSE) and the Young Mania Rating Scale (YMRS).

**Results:**

All groups demonstrated significant clinical improvements after the treatment period; however, the trends in MMSE scores showed no significant differences (*p* = 0.068). Notably, the reduction in YMRS scores significantly varied across groups (*p* < 0.001, effect size = 0.342), with the folate group demonstrating a significantly greater decrease compared to both the folate/B6 (*p* = 0.003) and placebo groups (*p* < 0.001). Recovery rates revealed that 80% of patients receiving folate showed over a 50% decrease in YMRS scores after 3 weeks, markedly higher than the other groups (*p* = 0.001).

**Conclusions:**

Our findings support the short‐term use of folate as a beneficial adjunct in treating acute manic episodes in bipolar I disorder. However, the addition of vitamin B6 did not yield additional advantages. These results may inform future treatment guidelines targeting acute mania in bipolar disorder, advocating for folate supplementation as a potential strategy to enhance therapeutic outcomes.

## Graphical abstract

Short‐term folate supplementation is a beneficial adjunct in treating acute manic episodes of bipolar I disorder, while vitamin B6 did not offer additional benefits. These findings may guide future treatment protocols, indicating folate supplementation as a potential strategy for enhancing therapeutic outcomes in acute mania.

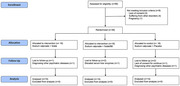

## 1 Introduction

Bipolar I disorder is a prevalent psychiatric condition, affecting approximately 1% of the global population over their lifetime. Individuals with bipolar I disorder experience debilitating episodes of mania, often exacerbated by insufficient treatment and inadequate follow‐up (Clemente et al. 2015). Each manic episode can adversely impact the mental well‐being of patients and their families, ultimately leading to a poor prognosis (Ahmed et al. 2021).

Pharmacotherapy remains the mainstay of treatment for bipolar I disorder. Mood stabilizers, such as lithium, carbamazepine, and sodium valproate, as well as atypical antipsychotics, are commonly prescribed to manage acute manic episodes (Nierenberg et al. 2023). However, the response rate to monotherapy is often less than 60% (Goodwin et al. 2009), and some patients continue to experience unresolved symptoms or adverse effects, highlighting the need to optimize treatment protocols. In recent years, there has been a growing interest in exploring the potential of nutrient‐based compounds as adjunctive therapies for psychiatric conditions, including bipolar disorder (Sienaert et al. 2013; Ashton et al. 2021).

In recent years, there has been a growing interest in exploring the potential of nutrient‐based compounds as adjunctive therapies for psychiatric conditions, including bipolar disorder (Sarris et al. 2022; Girone et al. 2023). Nutraceuticals may exert their effects by modulating various biological pathways implicated in the pathophysiology of bipolar disorder, such as neurogenesis, inflammation, oxidative stress, and mitochondrial function (Benmelouka et al. 2022; Wang et al. 2019). Examples of nutraceuticals investigated in prior studies include acetyl‐L‐carnitine, α‐lipoic acid, coenzyme Q10, creatine monohydrate, S‐adenosyl‐L‐methionine, folic acid, vitamin D, and N‐acetylcysteine (Burgess 2019; Rucklidge and Kaplan 2013).

Among the potential nutraceutical interventions, folate and vitamin B6 have garnered particular attention. Folate, a cofactor essential for nucleotide biosynthesis and neural function, has been proposed as a therapeutic agent for mood disorders due to its minimal adverse effects and potential to prevent mania‐like behaviors in animal models (Field and Stover 2018; Kronenberg et al. 2009; Hsieh et al. 2019; Brocardo et al. 2010). Research indicates a connection between folate deficiency and depression, with low folate linked to a higher risk of developing depression, more severe symptoms, longer episodes, and increased relapse rates. The primary neurobiological theory of major depressive disorder (MDD) involves monoamine neurotransmitters like serotonin (5HT), norepinephrine (NE), and dopamine (DA), which function within specific brain circuits. Folate is essential for regenerating tetrahydrobiopterin (BH4), a cofactor in neurotransmitter synthesis. Low folate can reduce levels of DA, NE, and 5HT, potentially increasing susceptibility to depression. However, this model requires further validation through additional research. Vitamin B6, in its coenzyme and metabolically active form, is mainly involved in the metabolism of amino acids. It is central in numerous reactions, such as transamination and decarboxylation. Vitamin B6 is also essential for the biosynthesis of neurotransmitters such as 5HT, epinephrine, NE, and GABA. The depressed phase of bipolar disorder consists of symptoms, including low energy, decreased hedonic capacity, and altered sleep and appetite, that fit into a model of 5HT signaling deficiency (Lan and John Mann 2016). Noradrenaline, DA, and GABA are also involved in 5HT signaling activation. Therefore, vitamin B6 has been suggested as a beneficial nutrient in the treatment of acute manic episodes (Dakshinamurti et al. 2014; Baradia et al. 2018; Massat et al. 2000).

While preliminary evidence suggests that adjunctive nutraceuticals may offer some utility in managing the depressive phase of bipolar disorder, the data remains inconclusive, and further investigation is warranted (Durga et al. 2007; Ma et al. 2016). Moreover, the supplementary role of folate and vitamin B6 in the treatment of mania in bipolar I disorder has been a subject of controversy (Calderón‐Ospina and Nava‐Mesa 2020; Danielski et al. 2018; Badrfam and Mostafavi 2021; Behzadi et al. 2009; Sánchez‐Villegas et al. 2009). Therefore, the present study aimed to evaluate the effectiveness of folate and vitamin B6 supplementation in the management of acute manic episodes in patients with bipolar I disorder.

## 2 Materials and Methods

### 2.1 Ethics

The study protocol was approved by the Ethics Committee of Mashhad University of Medical Sciences and was registered with the Iranian Clinical Trial Registry (IRCT201112188106N1). All participants or their legal guardians provided written informed consent after being informed about the intervention procedures and potential side effects.

### 2.2 Study Design

This was a randomized, double‐blind, placebo‐controlled trial that evaluated the efficacy of daily folate and folate/vitamin B6 supplementation in controlling acute manic episodes in patients with bipolar I disorder.

### 2.3 Study Population

Patients with a confirmed diagnosis of bipolar I disorder based on the *Diagnostic and Statistical Manual of Mental Disorders*, 4th ed., Text Revision (DSM‐IV‐TR) criteria were recruited from the Ibn‐e‐Sina Hospital of Psychiatric Disorders, Mashhad University of Medical Sciences, Mashhad, Iran.

### 2.4 Inclusion and Exclusion Criteria

Patients aged between 18 and 45 with a primary diagnosis of bipolar I disorder based on DSM‐IV‐TR, within an acute manic episode, whom a psychiatrist had initially interviewed via the Young Mania Rating Scale (YMRS), were included in the study. Moreover, a lack of history of a major mental illness, including schizophrenia, delirium, anorexia nervosa, bulimia nervosa, autism, mental retardation, attention deficit hyperactivity disorder, convulsion, drug dependence during the 3 months before the trial, being at risk of suicide or killing others, and previous psychiatric admissions during the 3 months before the trial, negative pregnancy test, lack of sensitivity to sodium valproate, and lack of consuming anti‐manic (mood stabilizing) medications 2 weeks before or during the trial were other inclusion criteria.

Patients were excluded from the study because of intolerance to increasing dosages of medications within a specified time and unwillingness to continue the study.

### 2.5 Randomization and Blinding

Of the 53 patients evaluated for eligibility, 45 were randomly assigned to one of three groups using a block randomization method. Each participant was identified by a unique three‐digit code, and the study medications (A: folate/vitamin B6, B: folate, or C: placebo) were placed in envelopes labeled with the corresponding codes. A researcher blinded to the group assignments delivered the medications to the participants. The participants, evaluators, analysts, and those responsible for group allocation remained blinded to the interventions throughout the study. However, the psychiatrist was aware of the treatment to monitor the effects and potential side effects.

### 2.6 Intervention

Patients were randomly assigned to one of three groups: (1) folate/vitamin B6 group, receiving 5 mg/day folate and 80 mg/day vitamin B6; (2) folate group, receiving 5 mg/day folate and two placebo tablets; and (3) placebo group, receiving three placebo tablets. All participants also received sodium valproate, starting at 600 mg/day and titrated up to 20–30 mg/kg or the maximum tolerated dose. The placebo was similar to the tablets of the intervention groups in terms of number, shape, color, and smell.

### 2.7 Study Procedure

This study consisted of 3–14 days of the basic phase (when the patient reaches a stable serum level) and a 4‐week maintenance period. During the basic stage, the patients were screened, which included a psychiatrist's interview and diagnosis of BMD1 with the last episode of acute mania, the YMRS test, determining the severity of symptoms based on YMRS, physical and neurological examination, laboratory evaluations, electrocardiography, and MMSE test. Also, if necessary, 2–4 mg of risperidone was prescribed for the patient. At this stage, a detailed description of recent treatments was obtained, and the criteria for entering the study were examined. Eligible patients got a three‐digit identification code, and a demographic questionnaire was filled out for all of them. Then, participants were placed in groups A, B, and C using the block randomization method. Group A was treated with sodium valproate and three placebos. Group B received sodium valproate, one 5 mg folic acid tablet, and two 40 mg vitamin B6 tablets. Group C was treated with sodium valproate, one 5 mg folic acid tablet, and two placebo tablets. In all three groups, sodium valproate was started with a dose of 600 mg daily and increased to 20–30 mg/kg or the highest amount the patient could tolerate. The therapeutic doses of folic acid and vitamin B6 were 5 and 80 mg daily, respectively. If the patient could not tolerate the speed of increasing the dose, the researcher could decrease it and bring it to the quorum until the end of the initial stage. However, after this stage, the dose was not allowed to be increased, and in case of intolerance, the patient was excluded from the study.

Considering the need to monitor the treatment's effects and the possibility of side effects, the psychiatrist knew the type of treatment but was not aware of the test results. The medical team was the same in all three groups to minimize the possibility of bias in sample selection, tests, patient follow‐up, and examination of the possibility of needing to withdraw from the study.

All study subjects underwent YMRS and MMSE at baseline and after the third and sixth weeks. Two psychiatrists evaluated the side effects of the administered medications at baseline and after the third and sixth weeks. Vital signs, such as pulse rate and blood pressure, were also measured three times a week during the trial.

### 2.8 Outcome Assessment

The primary outcome was the change in YMRS and MMSE scores from baseline to Week 6. The secondary outcome was defined as a 50% reduction in the YMRS score.

### 2.9 Instruments

#### 2.9.1 Young Mania Rating Scale

YMRS is one of the most important scales for measuring the severity of manic episodes (Young et al. 1978). It is an 11‐item, multiple‐choice diagnostic questionnaire used by a psychiatrist to determine the severity of manic episodes. It includes 11 criteria based on the patient's description of his/her clinical status during the 48 h before the evaluation. The criteria include elevated mood, increased motor activity/energy, sexual interest, sleep, irritability, speech (rate and amount), language/thought disorder, thought content, disruptive/aggressive behavior, appearance, and insight. In the Likert scoring of the YMRS, items are graded from 0 to 4 or 0 to 8 when the maximum grade is 60. Barakatain et al. (2007) validated YMRS for the Iranian population and determined Cronbach's alpha coefficient of internal consistency for patients and healthy individuals to be 0.73 and 0.63, respectively, and the test–retest reliability coefficient as 0.96. They also reported cut off, sensitivity, and specificity as 17.4, 98.4%, and 98.4% for the scale, respectively (Barakatain et al. 2007).

#### 2.9.2 Mini Mental Status Examination

It is mostly performed to screen for cognitive disorders and sometimes to investigate and monitor the effect of therapeutic interventions on patients' cognitive processes (Cockrell and Folstein 2002). In this research, MMSE was performed to screen patients; those with a total score of less than 20 were not included in the study.

### 2.10 Side Effect Evaluation

The safety of the drugs was evaluated by examining side effects, measuring vital signs such as pulse and blood pressure, and tests conducted based on suspicious clinical symptoms, ECG findings, and psychiatric and neurological examinations. Blood tests, serum biochemistry, and UA were performed in the basic phase and according to necessity every 2 weeks and at the end of the study. Physical and neurological examinations and ECG were performed at the baseline stage and the end of the sixth week. Severe side effects of administered medication, such as ataxia, dysarthria, disorientation, diplopia, and nystagmus, were monitored after the third and sixth weeks for all study subjects.

### 2.11 Sample Size Calculation

A previous study on folic acid add‐on treatment in patients with acute mania revealed a significant difference between YMRS scores after 4 weeks: 34.5 ± 3.9 vs. 30.1 ± 3.8 in control and intervention groups (Modabbernia et al. 2011). Considering alpha = 0.05 and beta = 0.2, each group's sample size was 14. With a drop‐up rate of 10%, the final samples accounted for 16 individuals in each group.

### 2.12 Statistical Analysis

The data were analyzed using SPSS v16 software. Descriptive statistics, chi‐square tests, and ANOVA (or its nonparametric equivalent) were used, with a significance level of 0.05.

## 3 Results

### 3.1 Baseline Characteristics of the Study Population

**FIGURE 1:**
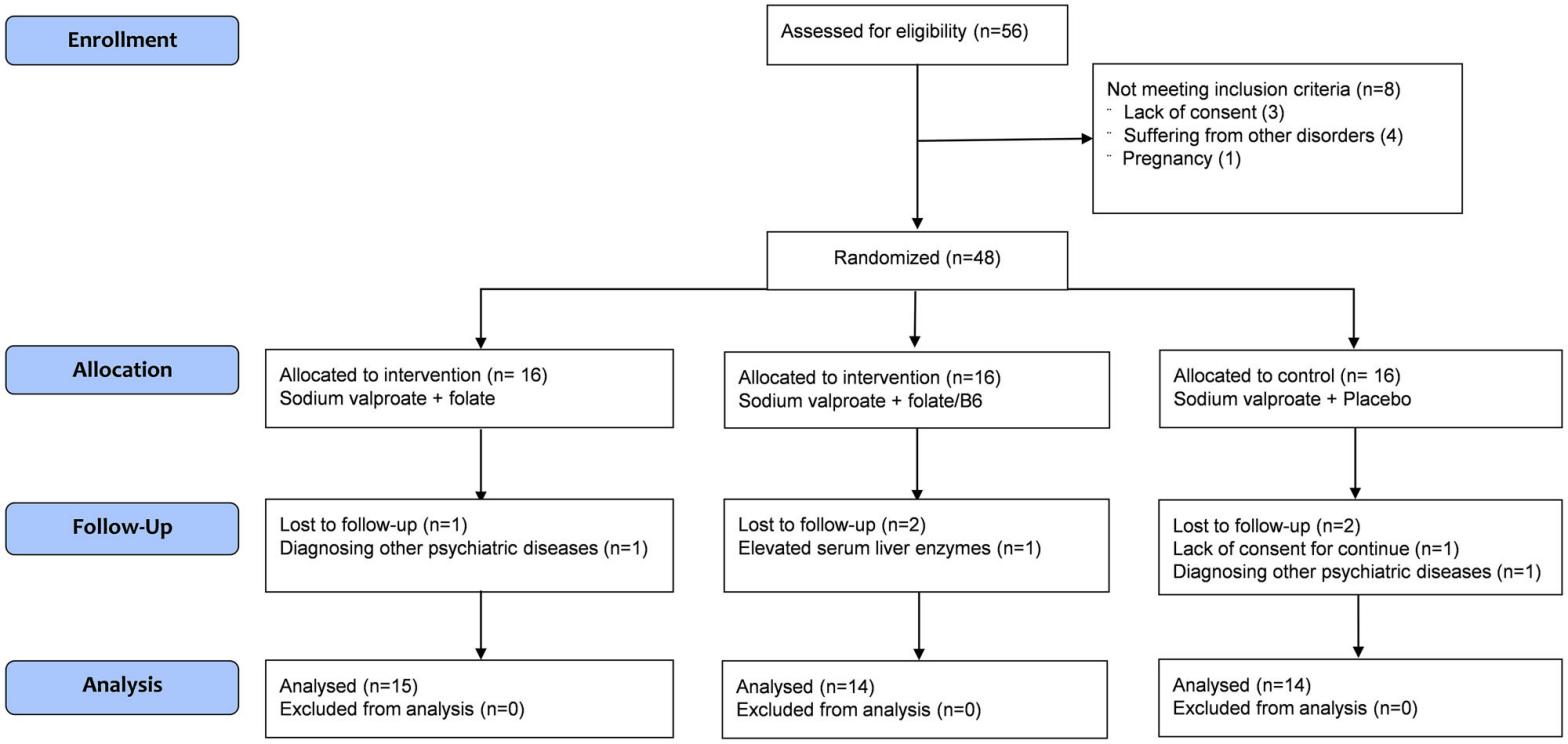
CONSORT diagram of the study population.

As shown in Figure 1, 56 patients with bipolar I disorder were evaluated for eligibility; of these, eight were excluded due to lack of consent or comorbidities. The remaining 48 patients were randomly assigned to the three treatment groups. Two patients in the placebo group withdrew due to lack of consent and the diagnosis of other psychiatric disorders. One patient in the folate group and another in the folate/vitamin B6 group were excluded due to the diagnosis of other psychiatric disorders and elevated liver enzymes, respectively. Ultimately, data from 43 participants (placebo, *n* = 14; folate, *n* = 15; folate/vitamin B6, *n* = 14) were included in the analysis.

### 3.2 Baseline Characteristics

Baseline demographic and clinical characteristics were similar across the three groups (Table 1). The mean age of the participants was 30.24 years, and there were no significant differences in age (*p* = 0.548), sex distribution (*p* = 0.479), smoking (*p* = 0.815), employment status (*p* = 0.497), and duration of illness (*p* = 0.871) between the groups.

**TABLE 1:** Study population characteristics.

Characteristics		Placebo (14)	Folate (15)	Folate/B6 (14)	*p* value
Age		29.36 ± 8.08	31.07 ± 6.82	32.64 ± 8.64	0.548
Sex	Male	8 (57.1%)	8 (53.3%)	9 (64.3%)	0.479
	Female	6 (42.9%)	7 (46.7%)	5 (35.7%)	
Disease duration	< 1	3 (21.4%)	4 (26.7%)	5 (35.7%)	0.871
	1–5	6 (42.9%)	5 (33.3%)	3 (21.4%)	
	> 5	5 (35.7%)	6 (40%)	6 (42.9%)	
Smoking	No	12 (85.7%)	13 (86.7%)	11 (78.6%)	0.815
	Yes	2 (14.3%)	2 (13.3%)	3 (21.4%)	
Occupation	Unemployed	8 (57.1%)	8 (53.3%)	11 (78.6%)	0.497
	Employed	6 (42.9%)	7 (46.7%)	3 (21.4%)	

### 3.3 Mini Mental Status Examination

**FIGURE 2:**
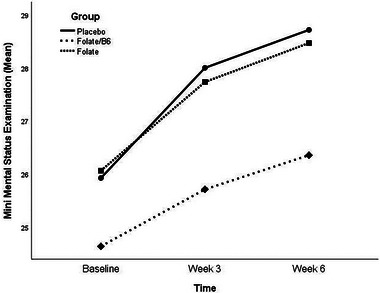
Comparing the change trend of MMSE scores during the trial between placebo, folate, and folate/B6 groups (*p* = 0.068).

The mean MMSE scores at baseline, Week 3, and Week 6 are presented in Table 2. At baseline, the scores were comparable among the three groups (*p* = 0.384). While the scores increased after 3 weeks, the difference between groups was not statistically significant (*p* = 0.064). However, the MMSE score increase differed significantly between groups after 6 weeks (*p* = 0.031). As shown in Figure 2, all groups experienced significant clinical improvement over the trial period, but the changing trend of MMSE scores did not differ significantly between groups (*p* = 0.068).

**TABLE 2:** Comparing **MMSE scores** between groups and different times of the study.

Time	Placebo (14)	Folate (15)	Folate/B6 (14)	*p* value*
Baseline	25.93 ± 2.09	26.07 ± 3.24	24.68 ± 3.50	0.384
Week 3	28.00 ± 2.35	27.73 ± 2.12	25.71 ± 3.56	0.064
Week 6	28.71 ± 2.16	28.47 ± 1.88	26.36 ± 3.27	**0.031**
*p* value	**< 0.001**	**0.001**	**0.002**	

^*^ Bold values indicate statistically significant results at p < 0.05.

### 3.4 Young Mania Rating Scale

Table 3 displays the YMRS scores at baseline, Week 3, and Week 6. The mean baseline YMRS scores were similar across the groups (*p* = 0.403). A significant decrease in YMRS scores was observed in the placebo (*p* < 0.001), folate (*p* < 0.001), and folate/vitamin B6 (*p* < 0.001) groups after 6 weeks. The YMRS score improvement was significantly different between groups at both Week 3 (*p* = 0.001) and Week 6 (*p* < 0.001). Post hoc analysis revealed that the YMRS improvement was significantly greater in the folate group compared to the folate/vitamin B6 (*p* = 0.001) and placebo (*p* = 0.016) groups after 3 weeks. However, the folate/vitamin B6 and placebo groups did not differ significantly (*p* = 1.00). After 6 weeks, the YMRS score decrease was significantly greater in the folate group compared to both the folate/vitamin B6 (*p* = 0.014) and placebo (*p *< 0.001) groups.

**TABLE 3:** Comparing **YMRS scores** between groups and different times of the study.

Time	Placebo (14)	Folate (15)	Folate/B6 (14)	*p* value*
Baseline	21.14 ± 1.95	22.00 ± 2.44	22.35 ± 2.79	0.403
Week 3	14.14 ± 4.31	8.26 ± 4.86	16.00 ± 6.63	**0.001**
Week 6	13.00 ± 5.21	3.13 ± 1.64	7.28 ± 3.51	**< 0.001**
*p* value	**< 0.001**	**< 0.001**	**< 0.001**	

^*^ Bold values indicate statistically significant results at p < 0.05.

**FIGURE 3:**
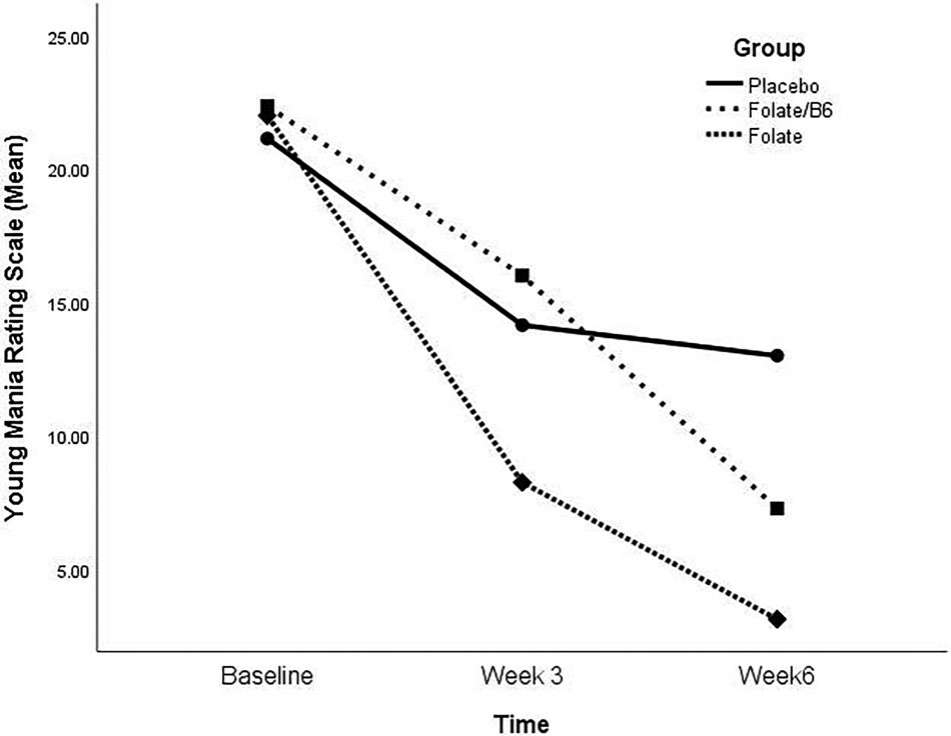
Comparing the change trend of YMRS scores during the trial between placebo, folate, and folate/B6 groups (*p* < 0.001); folate versus folate/B6 (*p* = 0.003), folate versus placebo (*p* < 0.001), folate/B6 versus placebo (*p* = 0.740).

As shown in Figure 3, the changing trend of YMRS scores over the trial period differed significantly between the groups (*p* < 0.001, effect size = 0.342). The YMRS decrease was significantly greater in the folate group compared to the folate/vitamin B6 (*p* = 0.003) and placebo (*p* < 0.001) groups, while the folate/vitamin B6 and placebo groups had a similar trend (*p* = 0.740).

Regarding the response rate (defined as a 50% or greater decrease in YMRS score), 12 patients (80.0%) in the folate group achieved this criterion after 3 weeks, which was significantly higher than the folate/vitamin B6 and placebo groups (*p* = 0.001). The response rate was 100% in the folate group and 85.7% in the folate/vitamin B6 group, compared to 21.4% in the placebo group (p < 0.001) (Table 4).

**TABLE 4:** Comparing complete recovery based on YMRS score between groups after 3 and 6 weeks.

50% recovery in YMRS score		Placebo (14)	Folate/B6 (14)	Folate (15)	*p* value
Week 3	No	11 (78.6%)	11 (78.6%)	3 (20.0%)	**0.001**
	Yes	3 (21.4%)	3 (21.4%)	12 (80.0%)	
Week 6	No	11 (78.6%)	2 (14.3%)	0 (0.0%)	**< 0.001**
	Yes	3 (21.4%)	12 (85.7%)	15 (100.0%)	

^*^ Bold values indicate statistically significant results at p < 0.05.

### 3.5 Safety Evaluation

No severe adverse events were reported during the trial. However, one patient in the folate/vitamin B6 group was excluded due to a three‐fold increase in serum liver enzyme levels.

## 4 Discussion

The present study aimed to evaluate the efficacy of adding folate or folate/B6 supplementation to sodium valproate in the management of acute manic episodes in patients with bipolar I disorder. The findings suggest that folate, but not the combination of folate and vitamin B6, had a positive impact on the severity of manic symptoms. However, neither folate nor folate/B6 supplementation demonstrated significant improvements in cognitive function.

The present study found no significant improvement in cognitive function among patients with bipolar I disorder following supplementation with folate or folate combined with vitamin B6. A thorough review of the literature revealed a lack of studies specifically examining the impact of folate on cognitive function in individuals with bipolar disorder. However, research on mild cognitive impairment has shown that daily oral administration of a 400 µg folic acid supplement for 12 months resulted in improved cognitive performance (Ma et al. 2016). Furthermore, a 3‐year folic acid supplementation study indicated positive effects on cognitive domains that typically decline with aging (Durga et al. 2007). Conversely, an intervention involving 8 weeks of vitamin B6 supplementation did not demonstrate any significant modification of cognitive function in bipolar I patients (Badrfam and Mostafavi 2021). Another study reported no evidence of short‐term benefits from vitamin B6 in enhancing mood (including symptoms of depression, fatigue, and tension) or cognitive functions (Malouf and Grimley Evans 2003). These findings suggest that the effectiveness of folate and vitamin B6 on cognitive performance may be dependent on both dosage and duration of supplementation. However, further clinical trials are necessary to validate their potential effectiveness.

Several studies have reported lower serum folate levels in patients with bipolar disorder compared to control subjects (Hsieh et al. 2019). Conversely, evidence suggests that adjunctive folate use may enhance the response to antidepressant medications (Fava 2007). These findings support the hypothesis that folate supplementation could be beneficial in managing manic episodes. The present study demonstrated a significant decrease in YMRS scores, indicating reduced mania severity in bipolar I patients following folate supplementation. Similarly, a prior study found a reduction in manic severity among bipolar patients treated with sodium valproate in conjunction with 3 mg of folate daily after 3 weeks, yielding an effect size of 0.3 (Behzadi et al. 2009). The positive impact of folate was also noted when added to lithium and risperidone in patients experiencing acute mania (Modabbernia et al. 2011). However, inconsistently, another study investigating the effects of adding lamotrigine and/or folic acid to quetiapine reported no superiority of folic acid over placebo. This study also found that folic acid may counteract the efficacy of lamotrigine on depressive symptoms (Geddes et al. 2016). Nonetheless, a systematic review positioned adjunctive folate as an effective and safe treatment for MDD and bipolar manic episodes, although it was not supported for schizophrenia (Zheng et al. 2020). The potential effectiveness of folate may be attributed to its critical role as a cofactor in DNA and RNA synthesis, methionine re‐methylation, gene expression, and protein synthesis. Given that studies have shown significant neuronal damage during acute manic episodes, which can lead to a reduction in cortical gray matter (Uribe and Wix 2012; Abe et al. 2015), the necessity for folate supplementation may arise from nutritional deficiencies resulting from suboptimal eating behaviors, allowing for recovery from these deficits.

Another finding of the present study was the lack of superiority of folate and vitamin B6 supplementation over placebo in managing manic episodes. However, the recovery rate was significantly higher in patients receiving folate and vitamin B6 compared to those on placebo. We hypothesize that vitamin B6 may counteract the effectiveness of folate in addressing mania, suggesting that the observed positive effect of the folate and vitamin B6 combination on recovery rates was primarily attributed to folate rather than vitamin B6. To the best of our knowledge, no studies have specifically investigated the effects of combining folate and vitamin B6 in the treatment of manic episodes. Nonetheless, two studies have evaluated the addition of vitamin B6 to lithium therapy in bipolar patients (Badrfam and Mostafavi 2021; Zandifar et al. 2024). A recent report indicated that adjunctive vitamin B6 therapy with lithium may lead to improvements in mood symptoms during manic episodes in individuals with bipolar disorder (Zandifar et al. 2024). Conversely, another investigation found no positive impact of vitamin B6 supplementation on manic symptoms after an 8‐week period (Badrfam and Mostafavi 2021). Molecular analyses suggest that plasma levels of vitamin B6 are reduced in individuals with depression (Ryan et al. 2020). In addition, B6 supplementation has shown benefits in alleviating depressive symptoms in young women using oral contraceptives (Johnston and Curtin 2020). These findings underscore the potential benefits of vitamin B6 in modifying depressive symptoms; however, its efficacy may be influenced by hormone‐related pathways, gender differences, and comorbid conditions.

As a strength, the present study was a controlled clinical trial consisting of two groups under supplementary therapy compared with placebo receivers. Although there was no previous report on folate/B6 supplementation for bipolar patients, and the study was designed as a pilot with low samples, the achieved power was more than 95%. However, some limitations should be acknowledged. A small sample can limit the ability to detect effects and might not capture the diversity of a larger population. Vitamin B6 alone was not considered, making it difficult to evaluate its effect on acute manic episodes. As there were no adverse effects on folic acid and vitamin B6 supplementation with the considered dosage, more follow‐up time may present more precise results. A brief follow‐up period may not adequately assess the long‐term effects of folate and vitamin B6 supplementation on manic episodes or cognitive function. Longitudinal studies could provide more insights into how these supplements affect mood and cognition over time. Also, the study did not explore a range of doses for folate and vitamin B6 as different dosages might yield different results. Understanding the biological pathways through which folate may affect mood stabilization could be insightful.

## 5 Conclusions

The current clinical trial provides evidence that folate supplementation, when added to sodium valproate, can improve the clinical outcomes of acute manic episodes in patients with bipolar I disorder, with minimal side effects. This finding supports the use of folate as an effective and safe adjunct pharmacotherapy for the management of mania in these patients. However, the combination of folate and vitamin B6 did not demonstrate superior efficacy compared to placebo. Further research is needed to explore the potential benefits, optimal dosing, and recovery mechanism of folate and vitamin B6, alone or in combination, in the treatment of bipolar disorder.

## Author Contributions

**Farzad Akbarzadeh**: methodology, data curation, writing – review and editing. **Andisheh Talaei**: methodology, formal analysis, writing – review and editing. **Mohsen Nemati**: supervision, conceptualization, writing – review and editing. **Dina Ganji**: data curation, methodology, writing – review and editing. **Alireza Ebrahimi**: conceptualization, methodology, data curation, writing – review and editing, writing – original draft. **Ali Talaei**: conceptualization, supervision, funding acquisition, project administration, writing – review and editing, methodology.

### Peer Review

The peer review history for this article is available at https://publons.com/publon/10.1002/brb3.70432.

## Data Availability

The data that support the findings of this study are available on request from the corresponding author. The data are not publicly available due to privacy or ethical restrictions.
